# BEST1 Positive Monocytes in Circulation: Visualize Intratumoral Crosstalk between Cancer Cells and Monocytes

**DOI:** 10.1002/advs.202205915

**Published:** 2023-04-23

**Authors:** Luyao Zhang, Yiran Wang, Wei Yuan, Changming An, Qin Tan, Jie Ma

**Affiliations:** ^1^ Center of Biotherapy Beijing Hospital National Center of Gerontology Institute of Geriatric Medicine Chinese Academy of Medical Sciences Beijing 100730 P. R. China; ^2^ State Key Laboratory of Molecular Oncology National Cancer Center/ National Clinical Research Center for Cancer/Cancer Hospital Chinese Academy of Medical Sciences & Peking Union Medical College Beijing 100021 P. R. China; ^3^ Department of Head and Neck Surgery National Cancer Center/ National Clinical Research Center for Cancer/Cancer Hospital Chinese Academy of Medical Sciences & Peking Union Medical College Beijing 100021 P. R. China

**Keywords:** bestrophin1, head and neck squamous cell carcinoma, monocytes, tumor microenvironment, VEGF‐A

## Abstract

Head and neck squamous cell carcinomas (HNSCCs) are characterized by an abundance of monocytes and macrophages recruited from the peripheral blood. However, it has not been determined whether these infiltrated cells can be released back into circulation with a tumor‐associated neobiosignature. This study reports that Bestrophin1 (BEST1), a component protein of Ca^2+^‐activated Cl^−^ channels (CaCCs), is highly expressed on classical monocytes in the peripheral blood of HNSCC patients. This is due to monocyte education by tumor cells, in which tumoral VEGF‐A upregulates BEST1 expression on monocytes through the MEK‐ERK‐ELK1 pathway. This leads to improved secretion of IL‐6 and IL‐8, which promotes tumor cell proliferation. This work also finds that BEST1 facilitates the motility of monocytes, contributing to the migration of these cells back into circulation. These results suggest that the expression of BEST1 on peripheral monocytes may be a potential tool for monitoring tumor progression, and opens up the possibility of searching for cancer biomarkers on monocytes rather than on the tumor or its products.

## Graphical abstract

Bestrophin1 expression on monocytes is elevated in head and neck squamous cell carcinoma patients and may serve as a potential biomarker for tumor progression. Monocyte education by tumor cells through VEGF‐A also enhances secretion of IL‐6 and IL‐8, promoting tumor cell proliferation.

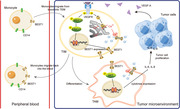

## 1 Introduction

Plenty of studies have shown that in response to tumor microenvironment (TME), myeloid‐derived monocytes in circulation can be recruited to the tumor, where a part of them differentiate into tumor‐associated macrophages (TAMs).^[^ ^1^, ^2^, ^3^ ^]^ An investigation of tumor infiltrated immune cells in tumor tissues revealed that head and neck squamous cell carcinoma (HNSCC), breast cancer, lung cancer and colon cancer had relatively high proportion of tumor infiltrated monocytes (TIM) as compared with other malignant tumors, which associated with cancer occurrence and prognosis in these tumors.^[^ ^4^ ^]^ Although it has been reached a consensus that immune cells play important role in TME, the crosstalk between TIM and tumor cells still needs to be further elucidated. This crosstalk might result in two aspects: first, promoting tumor cell proliferation or metastasis by TIM through the production of cytokines such as interleukin‐6 (IL‐6) and IL‐8;^[^ ^5^, ^6^ ^]^ second, modifying transcriptional landscape of TIM by tumor such as the specific expression of genes SIGLEC1 or STING1, which reflect the prognosis of patients to a certain extent.^[^ ^7^, ^8^ ^]^ In terms of shaping TIM, whether the impress of crosstalk on TIM can be detected in places other than tumor has not been disclosed since evidence of monocytes circulating back to the blood is few. We hypothesize that TIMs recruit back to the circulation after the crosstalk, the phenotype of peripheral monocytes will be rewritten. Therefore, the existence of cancer stamped immune cells in peripheral blood might provide as distinct immune cell signatures of cancer.

A gene BEST1 encodes Bestrophin1 (BEST1) protein, which is a member of the bestrophin family (BEST1‐4). This protein is expressed in the plasma membrane and forms Ca^2+^‐activated Cl^−^ channels (CaCCs) by assembling as pentamers.^[^ ^9^ ^]^ BEST1 was first identified as a pathogenic gene of Best's macular dystrophy (BMD).^[^ ^10^, ^11^ ^]^ Moreover, recent studies have shown that BEST1 can regulate chloride currents in respiratory and renal epithelial cells and promote the proliferation of tumor cells.^[^ ^12^, ^13^, ^14^ ^]^ However, at present, the function and mechanism of BEST1 in immune cells has not been thoroughly studied.

In this study, we report that BEST1 is highly expressed on classical monocytes in peripheral blood in HNSCC patients. The mechanism behind this is monocyte education by tumor cells. Precisely, tumoral VEGF‐A up‐regulates BEST1 expression on monocytes through the MEK1/2‐ERK1/2‐ELK1 pathway, which improves secretion of cytokines IL‐6 and IL‐8, leading to the promotion of tumor cell proliferation. Interestingly, in addition to the regulation of tumor cells, BEST1 facilitates the motility of monocytes, which contributes to the migration of educated monocytes back into circulation.

Conventional thinking drove to the search for liquid tumor biomarkers in tumor or its products,^[^ ^15^, ^16^ ^]^ but here we identified a potential marker in peripheral monocytes reflecting the existence of HNSCC. We hope our study opened a new window for searching cancer biomarkers on immune cells rather than on tumors and their products.

## 2 Results

### 2.1 BEST1 is Up‐Regulated in Peripheral Monocytes of HNSCC Patients

**Figure 1:**
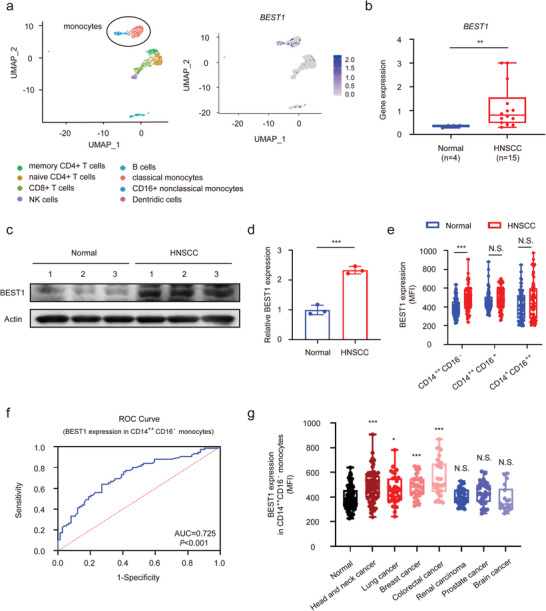
BEST1 is up‐regulated in peripheral monocytes of HNSCC patients. a) Uniform manifold approximation and projection (UMAP) plots of leukocytes overlaid with color‐coded clusters in peripheral blood from patients with HNSCC. Ellipse highlights two clusters of monocytes (Left). Feature plots of relative levels of *BEST1* expression on sorted peripheral blood leukocytes using scRNAseq analysis (Right). b) *BEST1* mRNA levels in peripheral blood monocytes from patients with HNSCC (*n* = 14) and health donors (*n* = 4) using scRNAseq analysis. c,d) Representative western blot (c) and associated quantifications (d) of BEST1 expression on CD14^+^ monocytes from patients with HNSCC (*n* = 3) and health donors (*n* = 3). e) Percentages for the expression of BEST1 on classical (CMs, CD14^++^, CD16^−^), intermediate (IMs, CD14^++^CD16^+^), and nonclassical (NMs, CD14^+^CD16^++^) monocytes in peripheral blood from patients with HNSCC (*n* = 75) and health donors (*n* = 74). f) Receiver operating characteristic (ROC) curve of BEST1 expression of classical monocyte as a diagnostic indicator of HNSCC from (e). The area under curve (AUC) calculated by R software OptimalCutpoints package was 0.725, *p* < 0.001. g) Percentages for the expression of BEST1 on classical monocytes in peripheral blood from patients with head and neck cancer (*n* = 75), breast cancer (*n* = 36), colorectal cancer (*n* = 32), lung cancer (*n* = 35), renal carcinoma (*n* = 38), prostate cancer (*n* = 34), brain cancer (*n* = 18), and health donors (*n* = 74). Data are represented as mean ± SD. Statistical significance is indicated by *p < 0.05, **p < 0.01, ***p < 0.001. N.S. = not significant; Student's *t*‐test (b,d,e); one‐way ANOVA (g).

To explore molecules specifically expressed in peripheral immune cells in HNSCC, we selected the scRNA‐seq data generated from peripheral blood mononuclear cells (PBMC) from 26 patients and 6 healthy donors (GSE139324).^[^ ^17^, ^18^ ^]^ We first analyzed the dataset by reducing the dimensionality of the dataset and visualizing the 8 lineage‐specific clusters via uniform manifold approximation and projection (UMAP) plots and assigned cell type identity to clusters with canonical markers (**Figure** **1**a). Next, we performed differentially expressed genes (DEGs) analysis of monocytes clusters (CD14^+^ or CD16^+^) in HNSCC patients and healthy donors, and obtained 9 genes highly expressed in patients group (Table S1, Supporting Information). We then analyzed these 9 genes’ expression on each cell cluster of each sample, and captured only one gene, BEST1, which was specifically highly expressed in identified monocytes (Figure 1a,b). To confirm the results of bioinformatics analysis, we examined BEST1 expression on peripheral blood monocytes from patients with HNSCC. Western blot analysis indicated an increased expression of BEST1 in monocytes of HNSCC patients (Figure 1c,d) after magnetically isolating the CD14^+^ monocytes from PBMC (Figure S1a, Supporting Information). Human monocytes can be subdivided into three subsets according to the expression of CD14 and CD16, that is, classical (CD14^++^CD16^−^), intermediate (CD14^++^CD16^+^), and nonclassical (CD14^+^CD16^++^).^[^ ^19^ ^]^ We found that BEST1 was expressed in all three subsets, and the expression level was significantly elevated on classical, but not intermediate or nonclassical monocytes from patients with HNSCC compared with those of healthy donors via flowcytometry (Figure S1b, Supporting Information and Figure 1e). According to the calculation of the expression level of BEST1 in classical monocytes, the area under ROC curve of BEST1 expression was 0.725, which revealed fairly good diagnosis of HNSCC (Figure 1f). These findings suggested that BEST1 could be used as a diagnostic indicator to distinguish between HNSCC patients and healthy donors.

We asked whether similar changes in blood classical monocytes also occurred in other common malignancies. Intriguingly, in addition to HNSCC, the lung, breast, and colorectal cancer, had consistently high levels of BEST1 expression on classical monocytes (Figure 1g). However, there was no significant difference of BEST1 expression in the renal, prostate, and brain cancer (Figure 1g). We further analyzed the immune infiltration, which was estimated by CIBERSORT algorithms, across diverse cancer types from The Cancer Genome Atlas (TCGA), and found that the different expression of BEST1 in peripheral blood monocytes of diverse cancers was consistent with the proportion of infiltration of monocytes and macrophages in tumor microenvironment (Figure S1c, Supporting Information). Altogether, these results suggested that BEST1 was up‐regulated in peripheral monocytes of HNSCC patients, which may be related to the high proportion of infiltration of monocytes in TME.

### 2.2 The Monocytes with High Expression of BEST1 in Peripheral Blood are Derived from the Release of Tumor‐Infiltration Monocytes

**Figure 2:**
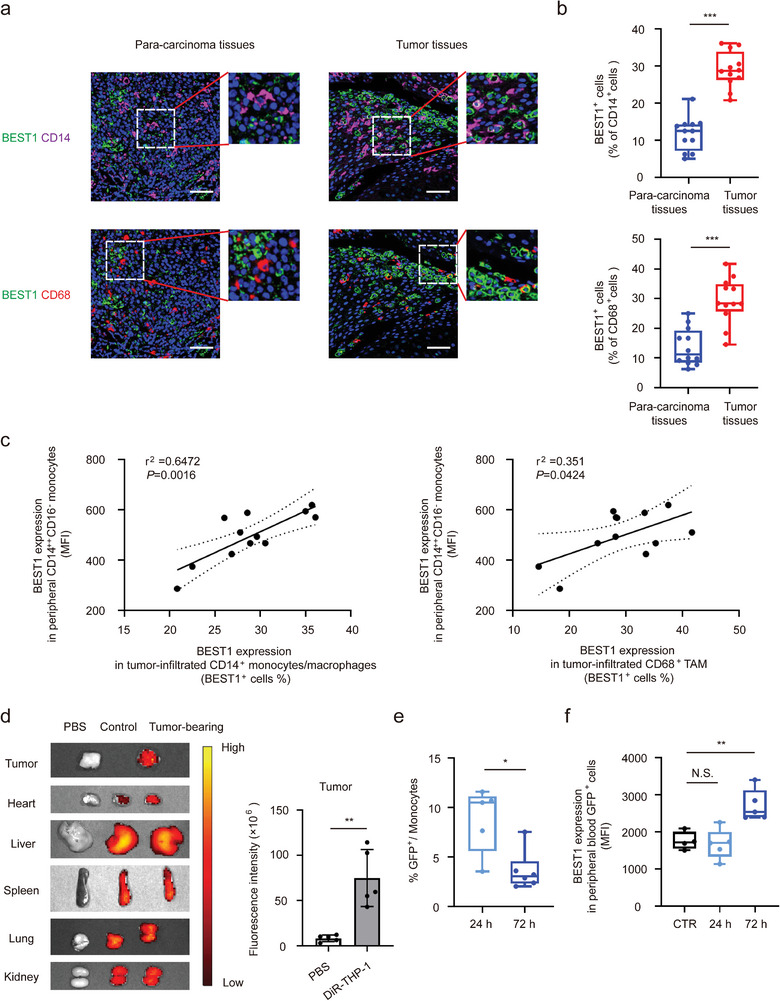
The monocytes with high expression of BEST1 in peripheral blood are derived from the release of tumor‐infiltration monocytes. a,b) Representative images (a) and quantification (b) of BEST1^+^ monocytes and macrophages in HNSCC tumors and adjacent para‐cancer tissues (*n* = 12 from one experiment representative of three independent experiments). Blue, DAPI; magenta, CD14; red, CD68; green, BEST1. Scale bar, 100 mm. c) BEST1^+^ cell ratio in tumor‐infiltrated CD14^+^monocytes/macrophages and CD68^+^ TAMs are associated with BEST1 expression in peripheral CD14^++^ monocytes (*n* =  12) (Pearson's correlation coefficient R and two‐tailed *p* values are shown). d) Representative ex vivo fluorescent images of the DiR‐labeled THP‐1 in tumors and other organs (*n* = 5 mice per group from one experiment representative of two independent experiments). e,f) GFP‐THP‐1 was injected into tumor‐bearing mice, after 24 or 72 h, the ratio of GFP^+^ cells (e) and BEST1 expression level (f) in peripheral blood monocytes by flow cytometry (*n* = 5 mice per group from one experiment representative of two independent experiments). Data are represented as mean ± SD. Statistical significance is indicated by *p < 0.05, **p < 0.01, ***p < 0.001. N.S. = not significant; Student's *t*‐test (b,d,e); one‐way ANOVA (f).

To explore the correlation between high expression of BEST1 monocytes in peripheral blood and tumor‐infiltrated monocytes, we performed scRNA‐seq data analysis of HNSCC tumor tissue (GSE103322)^[^ ^20^ ^]^ and paired carcinoma and adjacent tissue sections staining. We found that BEST1 was mainly expressed on monocytes (CD14^+^) and tumor‐associated macrophages (TAMs) (CD68^+^) in the TME of HNSCC patients (Figure S2a, Supporting Information), and the positive ratio of BEST1 expression in cancer is significantly elevated than that in adjacent normal tissue (**Figure** **2**a,b). Moreover, paired correlation analysis showed that the expression of BEST1 in peripheral blood monocytes strongly and positively correlated with the ratio of tumor infiltrated BEST1^+^ mononuclear phagocyte (Figure 2c), which prompted us to investigate the circulation of monocytes between peripheral blood and tumor.

Since monocytes can be recruited into the TME during tumorigenesis, and that in peripheral blood have transcriptional landscape related to tumor,^[^ ^1^, ^8^, ^21^ ^]^ we hypothesized that monocyte could increase the expression of BEST1 and could reflux back into peripheral blood after being influenced by TME. To trace monocyte trafficking route, DiR‐labeled human monocytes (THP‐1) were injected into mice bearing hypopharyngeal carcinoma tumor. 24 h after injection, tumor‐infiltrating monocytes were observed by ex vivo imaging (Figure 2d), and the distribution of monocytes in other organs were not affected by tumor‐bearing (Figure S2b, Supporting Information). We further performed intratumoral injection of GFP‐THP‐1 to investigate whether the tumor‐infiltrated monocytes could be released back into peripheral blood. We found these GFP labeled‐tumor‐infiltrating monocytes could be detected in peripheral blood both at 24 and 72 h after injection, and BEST1 expression was significantly increased on these monocytes at 72 h after injection (Figure 2e,f). These results delineated the circulation of monocytes between the peripheral blood and the tumor, that was, monocytes recruited into the TME to be obtained increased BEST1 expression and return back to peripheral blood subsequently.

### 2.3 Tumor Cells Induce BEST1 Expression on Monocytes and TAMs

**Figure 3:**
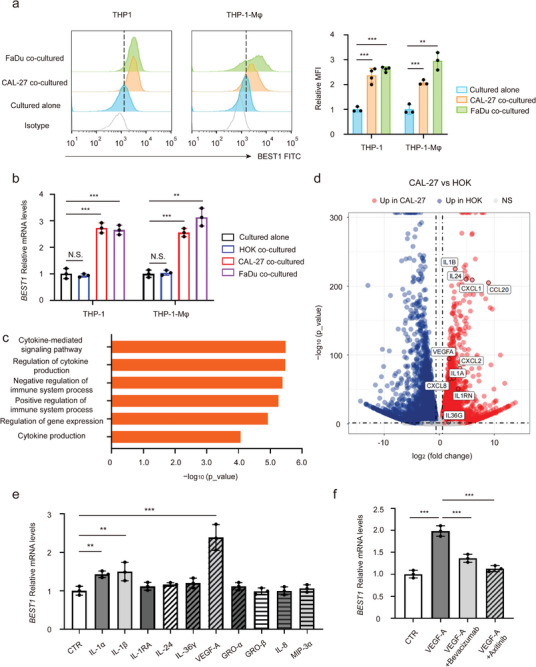
Tumor cells induce BEST1 expression on monocytes and TAMs. a,b) THP‐1 cells/THP‐1‐derived macrophages (THP‐1‐M*φ*) were cocultured with tongue squamous carcinoma cells CAL27, pharynx squamous carcinoma cells FaDu, or normal oral squamous epithelial HOK cells for 3 days. a) Representative histograms and summarized MFI for the expression of BEST1 by flow cytometry on THP‐1 and THP‐1‐M*φ* (*n* = 3 per group from one experiment representative of three independent experiments). b) Relative *BEST1* mRNA levels in THP‐1 and THP‐1‐M*φ*. The data are normalized to GAPDH (*n* = 3 per group from one experiment representative of three independent experiments). c) MetaCore Gene Ontology processes analysis displaying the processes associated with cytokines of CAL27 cells. d) Volcano plot showing the up‐ and down‐regulated transcripts by RNA sequencing in CAL27 compared to HOK cells (*n* = 3 per group). e) Relative *BEST1* mRNA levels in THP‐1 treated with 10 ng mL^−1^ cytokines for 24 h, respectively (*n* = 3 per group from one experiment representative of three independent experiments). f) After pretreatment with the humanized monoclonal antibody to VEGF‐A, Bevacizumab (0.2 µg mL^−1^), and the multi‐targeted tyrosine kinase inhibitor for VEGFR, Axitinib (5 nm) for 12 h, THP‐1 cells were stimulated with VEGF‐A for 24 h. Relative BEST1 mRNA levels in THP‐1 (*n* = 3 per group from one experiment representative of three independent experiments). Data are represented as mean ± SD. Statistical significance is indicated by **p < 0.01, ***p < 0.001. N.S. = not significant; one‐way ANOVA (a,b,e,f).

The evidence that BEST1 expression of monocytes associated with tumor education led to our exploration of the mediating mechanism. After co‐cultivating with HNSCC cell lines, the expression levels of BEST1 were significantly increased in both THP‐1 and THP‐1‐derived macrophages (THP‐1‐M*φ*) (**Figure** **3**a,b). Whereas, no significant change in BEST1 expression was detected when THP‐1 co‐cultured with normal oral squamous epithelial HOK cells, (Figure 3b). Generally, macrophages grow into two main groups called classically activated macrophages (M1) and alternatively activated macrophages (M2). M2 and a small fraction of M1 cells, also known as tumor‐associated macrophages (TAMs), facilitate proliferation and metastasis of tumor cells.^[^ ^4^ ^]^ Here we observed that co‐cultured macrophages mainly showed M2 signature through detecting characteristic markers (Figure S3a, Supporting Information). These results indicated that supernatant of HNSCC cells could promote BEST1 expression on monocytes and TAMs‐liked THP‐1, especially M2.

We further sought to investigate the factors in the supernatant that increased BEST1 expression on monocytes. Thus, we performed RNA sequencing and comparative analysis of human tongue squamous CAL27 cells and normal control HOK cells. Specifically, we shortlisted the significantly different genes (*p* < 0.05) for pathway and gene ontology (GO) analyses and considered pathways and terms significantly deregulated or enriched when *p* < 0.05. “Regulation of cytokine production” and “Cytokine‐mediated signaling pathway” were among the enriched terms identified by MetaCore GO processes (Figure 3c and Figure S3b, Supporting Information). We then profiled all cytokines and chemokines with significantly elevated expression in CAL27 and screened out 10 cytokines highly expressed in both two HNSCC cell lines by qPCR confirmation (Figure 3d and Figure S3c, Supporting Information). Next, different cytokines were used to stimulate THP‐1, respectively and we found that both VEGF‐A, IL‐1*α*, and IL‐1*β* could increase the expression of BEST1, where the effect of VEGF‐A is the most significant (Figure 3e). These high concentrations of cytokines could be detected in the supernatant of CAL27 and FaDu (Figure S3d, Supporting Information).

VEGF‐A intracellular signaling activities are mediated by the activation of VEGF receptors (VEGFR‐1, VEGFR‐2) which can be expressed on endothelial cells, tumor cells, and some immune cells.^[^ ^22^, ^23^ ^]^ We found that VEGFR‐1 and VEGFR‐2 were readily detected on THP‐1 monocytes (Figure S3e, Supporting Information). Interestingly, VEGF‐A‐mediated elevated BEST1 expression could be inhibited by anti‐VEGF‐A monoclonal antibody Bevacizumab or VEGFR tyrosine kinase inhibitor Axitinib (Figure 3f). These data suggested tumor cells‐derived VEGF‐A was indeed an important cytokine in inducing the BEST1 expression of mononuclear phagocytes.

### 2.4 VEGF‐A Regulates the Expression of BEST1 in Monocytes and TAMs through Transcriptional Activation of MEK‐ERK1/2‐ELK1 Pathway

**Figure 4:**
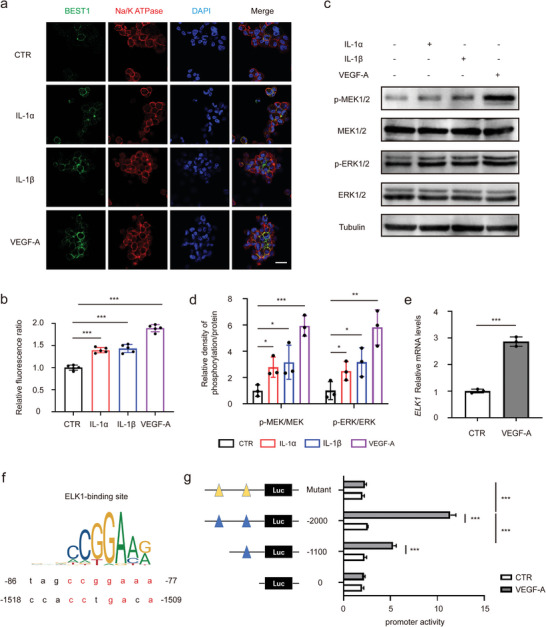
VEGF‐A regulates the expression of BEST1 in monocytes and TAMs through transcriptional activation of MEK‐ERK1/2‐ELK1 pathway. a,b) Representative immunofluorescence microscopy images (a) and quantification (b) of BEST1 and Na^+^‐K^+^ ATPase localization and expression in THP‐1 treated with 10 ng mL^−1^ cytokines for 24 h, respectively (*n* = 5 per group from one experiment representative of three independent experiments). c,d) THP‐1 cells were treated with 10 ng mL^−1^ cytokines for 15 min. Representative Western blots (c) and the associated quantifications (d) show phosphorylated MEK and ERK levels in cell lysates (Data are representative of three independent experiments). e) Relative *ELK1* mRNA levels in THP‐1 treated with 10 ng mL^−1^ VEGF‐A for 24 h. The data are normalized to GAPDH (*n* = 3 per group from one experiment representative of three independent experiments). f,g) THP‐1 cells were transfected with luciferase (Luc) reporter plasmids containing truncated and mutated BEST1 promoter and stimulated with PBS or VEGF‐A for 24 h, followed by luciferase reporter assays (*n* = 4 per group from one experiment representative of two independent experiments). Data are represented as mean ± SD. Statistical significance is indicated by *p < 0.05, **p < 0.01, ***p < 0.001; Student's *t*‐test (e); one‐way ANOVA (b,d); two‐way ANOVA with Tukey's test (g).

We further performed immunostaining to examine the subcellular localization of BEST1 and the plasma membrane marker Na/K‐ATPase. And we found BEST1 was indeed expressed on membrane (co‐localized with cytoplasmic membrane marker Na/K‐ATPase), as previously reported^[^ ^24^ ^]^ (**Figure** **4**a). The expression of BEST1 was significantly increased by cytokines stimulation, especially by VEGF‐A (Figure 4a,b). In addition, we also extracted the cell membrane protein to examine BEST1 expression, and the results were consistent with immunofluorescence (Figure S4a,b, Supporting Information). Once VEGF‐A interacts with VEGFR, its downstream phosphorylation cascade is activated to regulate gene expression.^[^ ^25^, ^26^ ^]^ Thus, we detected the MAPK pathway activity in monocytes. We found that the phosphorylation of MEK1/2 and ERK1/2, the key molecules in the MAPK pathway, were significantly increased after VEGF‐A stimulation (Figure 4c,d). IL‐1*α* and IL‐1*β*, which could also promote BEST1 expression, had slight activation effect. Moreover, ELK1, an ERK1/2 activated transcriptional factor, was also upregulated (Figure 4e). Sequence analysis revealed an ELK1‐binding site at −86 to −77 bp and −1518 to −1509 bp upstream of the BEST1 transcription start site (TSS) (Figure 4f). To further explore the transcription mechanism, THP‐1 was electronically transfected with a series of p GL4.10 reporter plasmids, which contained a sequential deletion of the 5′‐flanking region upstream of BEST1 and binding site mutant, and stimulated with VEGF‐A. We observed that deletion to −1100 bp and mutant, which were lack one of the ELK1 binding sites, significantly suppressed luciferase activities induced by VEGF‐A (Figure 4g). These results demonstrated that VEGF‐A in the tumor microenvironment increased BEST1 expression in monocytes and TAMs through MEK‐ERK1/2‐ELK1 signaling.

### 2.5 BEST1 Chloride Channels Enhance Motility and Migration of Monocytes and Macrophages

**Figure 5:**
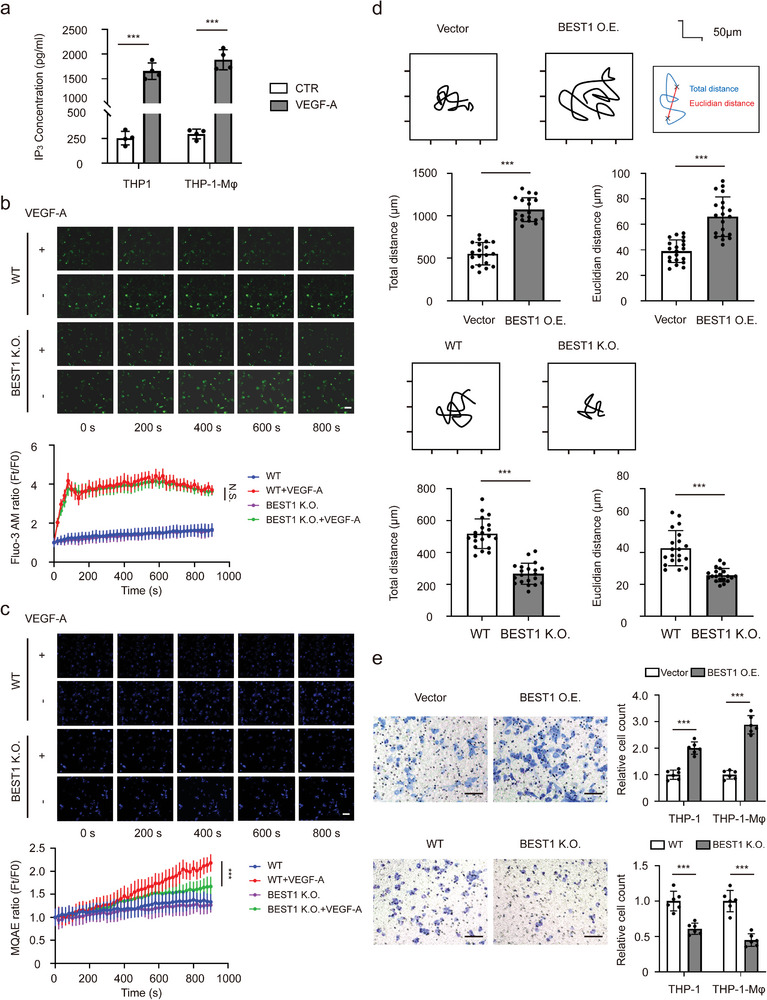
BEST1 chloride channels enhance motility, and migration of monocytes and macrophages. a) THP‐1 cells/THP‐1‐derived macrophages (THP‐1‐M*φ*) were treated with 100 ng mL^−1^ VEGF‐A for 15 min. IP_3_ levels in cell lysates from THP‐1 and M*φ* detected by ELISA (*n* = 3 per group from one experiment representative of two independent experiments). b,c) Representative immunofluorescence microscopy images and quantification of Fluo‐3AM (green) (b) and MQAE (blue) (c). Scale bar, 20 µm. (*n* = 3 per group from one experiment representative of three independent experiments). d) Representative 12 h time‐lapse tracking plots from the blank vector (Vector) and BEST1 expression (BEST1 O.E.) (upper), or wild‐type (WT) and BEST1 CRISPR‐Cas9 (BEST1 K.O.) (lower) THP‐1 cells. Corresponding histograms quantifying motility parameters (total distance traveled per cell) (*n* = 20 per group from one experiment representative of three independent experiments). e) Transwell cell migration assays in THP‐1 and M*φ* with Vector and BEST1 O.E. (upper), or WT and BEST1 K.O. (lower) (*n* = 6–8 per group from one experiment representative of two independent experiments). Data are represented as mean ± SD. Statistical significance is indicated by **p < 0.01, ***p < 0.001; Student's *t*‐test (a,d,e); two‐way ANOVA with Tukey's test (b,c).

Since BEST1 composes calcium‐activated chloride channels (CaCCs), we further investigated whether this membrane BEST1 has CaCC function in monocytes and TAM. The Ca^2+^ required for CaCCs opening could be obtained by intracellular calcium mobilization. Thus, molecules of several growth factors activate intracellular inositol 1,4,5‐triphosphate (IP_3_), which in turn binds to its receptor and initiates releasing of Ca^2+^ stored in the endoplasmic reticulum into the cytoplasm.^[^ ^27^, ^28^, ^29^ ^]^ VEGF‐A, one of such growth factors, is abundant in HNSCC tumor environment. The exposure to VEGF‐A resulted in high IP_3_ concentration in both THP‐1 and THP‐1‐derived macrophages (**Figure** **5**a).

To further explore the effect of BEST1 on the function of monocytes and macrophages, we established BEST1 stable expression, and BEST1 stable knockout (CRISPR/Cas9) THP‐1 cell lines (Figure S5a,b, Supporting Information). We then monitored intracellular Ca^2+^ and Cl^−^ by fluorescence probes Fluo‐3 AM and MQAE with live cell imaging in time‐course. In detail, both wild‐type (WT) and BEST1 KO THP‐1 cells were loaded with Fluo‐3 AM (Ca^2+^ imaging) or MQAE (Cl^−^ imaging) fluorescent probe. After that, cells were incubated with VEGF‐A at the concentration of 100 ng mL^−1^ and the fluorescence signal of probes were measured every 20 s for 15 min. We found that VEGF‐A addition caused rapid increase of intracellular Ca^2+^, followed by a sustained plateau, while caused decrease of intracellular Cl^−^ subsequently in WT THP‐1 (Figure 5b,c). However, in BEST1 KO THP‐1, VEGF‐A also induced intracellular Ca^2+^ release, but Cl^−^ efflux was significantly inhibited (Figure 5b,c). These results indicated that BEST1 played a key role in regulation of VEGF‐A‐mediated intracellular Cl^−^ efflux.

To confirmed activation of BEST1 by IP_3_‐mediated Ca^2+^ release, we treated THP‐1 cells with shRNA against ITPR1 to construct ITPR1 knockdown (KD) cells which with partially impaired IP_3_ receptor function.^[^ ^30^ ^]^ Besides, we transient overexpressed BEST1 for 48 h in ITPR1 KD cells to obtain ITPR1 KD cells with BEST1 overexpression. We then measured VEGF‐A‐stimulated intracellular calcium and chloride in these cells. In detail, WT, ITPR1 KD and ITPR1 KD with BEST1 overexpression THP‐1 cells were loaded with Fluo‐3 AM or MQAE fluorescent probe. After that, cells were incubated with VEGF‐A at the concentration of 100 ng mL^−1^ and the fluorescence signal of probes were measured every 20 s for 10 min. We observed decreased VEGF‐A‐induced Ca^2+^ mobilization, and decline of intracellular Cl^−^ was blocked in ITPR1 KD cells (Figure S6a,b, Supporting Information). However, inhibition of Cl^−^ efflux was slightly relieved when BEST1 expression was elevated in ITPR1 KD cells (Figure S6a,b, Supporting Information). These results revealed that IP_3_ combined with IP_3_R on the surface of the endoplasmic reticulum (ER) to release calcium stored in the ER into the cytoplasm, as a results, intracellular calcium facilitated chloride efflux via activating BEST1 composed CaCCs.

It has been reported that CaCCs played an important role in the process of cell motility and migration.^[^ ^27^, ^31^ ^]^ The involvement of the CaCCs in the process of cell morphological change is closely related to cell migration and invasion.^[^ ^32^, ^33^, ^34^ ^]^ Therefore, we performed live cell time‐lapse imaging to detect the tracking plots from BEST1 overexpression (O.E.), BEST1 knockout (K.O.), and their control (Vector, WT) THP‐1 cells. The results showed that over‐expressed BEST1 could enhance THP‐1 cells motility, while silencing of BEST1 reduced it (Figure 5d). This might contribute to the migration of monocytes back to peripheral blood. Furthermore, transwell assays reconfirmed this enhanced migration ability of monocytes and macrophages by BEST1 (Figure 5e). These data indicated that BEST1‐dependent chloride channels enhanced motility and migration of monocytes and macrophages.

### 2.6 BEST1 on TAMs Promotes the Development of Tumors via Secreting Cytokines IL‐6 and IL‐8

**Figure 6:**
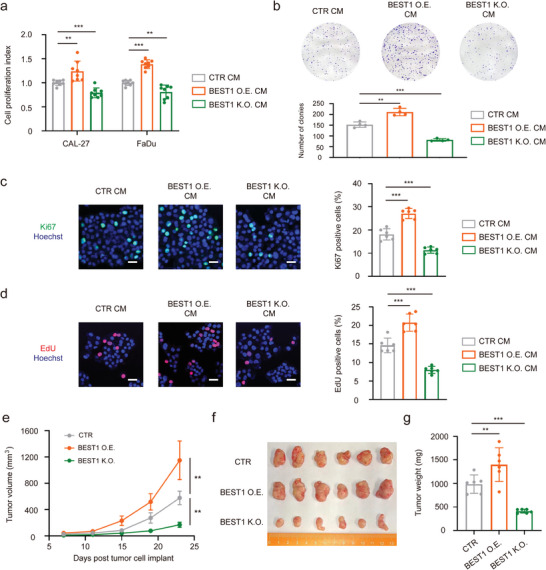
BEST1 on TAMs promotes the development of tumors via secreting cytokines. a) Cell growth for 48 h after the CAL27 and FaDu cells were cultured in the THP‐1 conditioned medium (CTR CM), THP‐1 with BEST1 O.E. conditioned medium (BEST1 O.E. CM), and THP‐1 with BEST1 K.O. conditioned medium (BEST1 K.O. CM) (*n* = 8 per group from one experiment representative of two independent experiments). b) Colony formation assay and for FaDu cells cultured in CTR CM, BEST1 O.E. CM, and BEST1 K.O. CM. c,d) Representative immunofluorescence microscopy images of Ki67 (green) (c) and Edu (red) (d) for FaDu cells cultured in CTR CM, BEST1 O.E. CM, and BEST1 K.O. CM. (*n* = 6 per group from one experiment representative of two independent experiments). e–g) 5‐week‐old nude mice were engrafted with FaDu cells combined with THP‐1 (CTR, BEST1 O.E., and BEST1 K.O.) at a 1:5 ratio (*n* = 6 mice per group). e) After 7 days, tumor volumes were calculated by measuring the length and width using Vernier calipers every 4 days. f) Images of the tumors from (e). g) Quantification of the tumor weights from (e). Data are represented as mean ± SD. Statistical significance is indicated by **p < 0.01, ***p < 0.001; one‐way ANOVA (a–d,g) two‐way ANOVA with Tukey's test (e).

Previous studies showed that tumor‐associated monocytes and macrophages represented the most abundant innate immune population in the tumor microenvironment (TME), even approaching to approximately 50% in some tumor types,^[^ ^35^ ^]^ we further investigated the function of BEST1 on tumor‐associated macrophages (TAM) for tumor progression. We cultured HNSCC cell lines with the conditioned medium (CM) of BEST1 O.E., BEST1 K.O. macrophages to determine tumor cells proliferation. The CCK8 assays showed that the BEST1 O.E. CM prompted the tumor cells proliferation (**Figure** **6**a), which were in line with the increased number of clone formation (Figure 6b). The increased number of Ki67 and EdU positive cells were increased in BEST1 O.E. CM treated cells, indicating the BEST1‐related proliferation and division (Figure 6c,d). Conversely, the cell proliferation and mitotic activity were inhibited by BEST1 K.O. CM accordingly (Figure 6b–d). Furthermore, to examine the impact of macrophage BEST1 on tumor development in vivo, a mixture of THP‐1 with BEST1 overexpression or knockout and FaDu cells at a ratio of 1:5 was implanted subcutaneously to the flanks of nude mice. Tumor growth status demonstrated that BEST1 on macrophages promotes tumor development in vivo (Figure 6e–g).

Several studies have reported that TAMs could secrete cytokines to promote tumor development, including IL‐6 and IL‐8.^[^ ^36^, ^37^, ^38^ ^]^ Here we attempted to ascertain whether BEST1 affects the cytokine secretory function of macrophages, which finally affects the tumor progression. We found an increased IL‐6 and IL‐8 secretion in BEST1 O.E. macrophages, which were significantly decreased in BEST1 K.O. macrophages. (Figure S7a,b, Supporting Information). To further investigate the transcriptional regulation mechanism of IL‐6 and IL‐8, we detected their common transcriptional regulator HIF‐1*α* and upstream AKT pathway.^[^ ^39^, ^40^, ^41^ ^]^ We found that AKT phosphorylation and HIF‐1*α* expression were elevated in BEST1 O.E. and reduced in BEST1 K.O. macrophages (Figure S7c,d, Supporting Information). These data demonstrated that BEST1 facilitated TAMs IL‐6, IL‐8 production to promote the development of tumors.

## 3 Discussion

**Figure 7:**
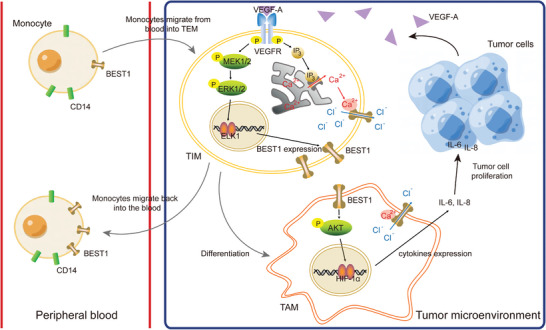
Schema depicting BEST1 positive monocytes/macrophages in circulation and TME. CD14^+^ monocytes migrate from blood into TME, where VEGF‐A induced the BEST1 expression of TIMs via MEK‐ERK1/2‐ELK1 transcriptional regulation. BEST1 facilitates the secretion of IL‐6 and IL‐8 in TAMs, leading to the promotion of tumor cell proliferation. In addition to the regulation of tumor cells, BEST1 also facilitates the motility of monocytes, which contributes to the migration of monocytes back into circulation.

Our study was initiated by a clinical discovery of the BEST1 highly expressed monocytes in peripheral blood from HNSCC patients. The interest to disclose the mechanism of this novel subset in association with tumor diagnosis led us to explore the crosstalk between tumor cells and monocytes in TME. Here, we highlighted BEST1 as a functional impress of the crosstalk between tumor cells and TIMs. We described how HNSCC tumor cells affect the expression, regulation, and function of chloride channel protein BEST1 in TIMs (**Figure** **7**).

BEST1 is expressed broadly, such as on the retinal pigment epithelium, epithelium of respiratory tract, neuron, and tumor cells.^[^ ^12^, ^14^, ^15^, ^42^ ^]^ However, there are no reports, to date, about BEST1 in study of immune cells. In our research, we identified BEST1 as a marker gene in peripheral blood monocytes of the HNSCC cohort dataset (GSE139324), which can potentially monitor tumor progression. However, we may not exclude other marker genes specifically expressed in tumor monocytes after expanding the sample size or analyzing other cancer types. The innovation of our research is the discovery of a BEST1 upregulated subset in peripheral monocytes in HNSCC patients, which is resulted from cancer education through VEGF‐A secretion. Besides playing an important role in the chemotaxis of tumor‐infiltrated monocytes, VEGF‐A stimulates autophosphorylation and downstream signal transduction of VEGFR‐1 and VEGFR‐2 in monocytes.^[^ ^26^, ^43^, ^44^ ^]^ Once VEGFR is activated, MAPK pathway is activated to regulate BEST1 gene expression, as well as IP_3_ induction to facilitate endoplasmic reticulum calcium store release. This made BEST1 expression meaningful because BEST1 is the core component protein of Ca^2+^‐ dependent chloride channels.^[^ ^45^, ^46^ ^]^ VEGF‐A induced rapid increase of intracellular Ca^2+^, followed by a sustained plateau,^[^ ^47^ ^]^ as a result, the IP_3_‐mediated Ca^2+^ caused BEST1 activation and decrease of intracellular Cl^−^ subsequently.^[^ ^14^, ^28^ ^]^ It has been reported that BEST1 had an ability to facilitate the movement of cells through regulation of cell volume.^[^ ^48^, ^49^ ^]^ In our in vivo or in vitro observations, similar phenomenon indicated that overexpression of BEST1 on TIMs enabled them to return to the peripheral blood. This could explain the clinical observation of the consistent existence of BEST1 positive monocytes in peripheral blood and tumor tissue.

In our study of the interacting proteins and downstream signaling molecules of BEST1, we observed that BEST1 expression activates AKT signaling to promote IL‐6 and IL‐8 production, which is consistent with several other studies.^[^ ^50^, ^51^, ^52^ ^]^ However, the specific mechanism needs to be further studied, which do not exclude the possibility that BEST1 promotes tumor cell proliferation beyond cytokines. In addition, the mechanisms underlying BEST1‐induced AKT/HIF‐1alpha activation has not been thoroughly studied in this paper, which is also the main content of our next research. The mechanism may also elicit a new intervention target for tumor suppression.

Finding cancer specific biomarkers for treatment and diagnosis has been challenging mankind for decades. Although collecting serum derived tumor‐associated protein are regarded as an effective method,^[^ ^53^ ^]^ dilution by the body fluid restricts its sensitivity especially at early stage of disease. Thus, detection of protein expression on peripheral blood cells might be a more accurate method with improved efficiency.^[^ ^54^ ^]^ Immune cells perform as information collectors and amplifiers in such kind of detection. Here in our study, we found BEST1 was elevated on monocytes in HNSCC, different from the previous intracellular detectable proteins such as TKTL1.^[^ ^55^ ^]^ Surface vestige of tumor education on the most abundant classical monocytes could be easily detected by flow cytometry, which made the detection easily to be adapted into clinical application.

Thus, the most important contribution of this study is to change the inherent mode of seeking biomarkers in cancer compartments or products. The discovery of BEST1 positive monocytes as a potential biomarker for HNSCC implied a new path to search cancer biomarkers.

## 4 Experimental Section

### Human Samples

The blood from patients with head and neck, lung, colon, breast, kidney, and brain cancers and healthy donors were collected at the Beijing Hospital and Cancer Hospital, Chinese Academy of Medical Sciences. The tumor tissues of HNSCC were collected at the Cancer Hospital, Chinese Academy of Medical Sciences. The use of pathological specimens, as well as the review of all pertinent patient records, were approved by the Ethics Committee of National Cancer Center/Cancer Hospital, Chinese Academy of Medical Sciences and Peking Union Medical College (project approval number 21/329‐3000).

### Mice

4‐week‐old BALB/c nude mice were purchased from the Beijing Hfk Bioscience Co., Ltd (Beijing, China) and housed in a pathogen‐free environment under a 12 h light‐dark cycle with free access to food and water in Cancer Hospital, Chinese Academy of Medical Sciences. All mouse protocols and experiments were performed in accordance with National Institutes of Health guidelines and were approved by the Ethics Committee for Animal Research of Cancer Hospital, Chinese Academy of Medical Sciences (project approval number NCC2021A277).

### Reagents

IL‐1RA (C033), IL‐36 (CM77), and VEGF165 (C083) were purchased from Novoprotein. CXCL1 (300‐11), CXCL2 (300‐39), CXCL8 (200‐08), IL‐1*α* (200‐01A), IL‐1*β* (200‐01B), IL‐24 (200‐35), TL‐1A (310‐23), and CCL20 (300‐29A) were purchased from Peprotech. Bevacizumab (HY‐P9906), and Axitinib (HY‐10065) were purchased from MedChemExpress.

### Cell Culture

All of the cell lines were cultured at 37 °C in a cell incubator with 5% CO2. THP‐1 cells were maintained in RPMI­1640 medium supernatant with 10% (v/v) FBS and 1% (v/v) pen–strep. CAL27 cells and HOK cells were maintained in DMEM medium supernatant with 10% (v/v) FBS and 1% (v/v) pen–strep. FaDu cells were maintained in MEM medium supernatant with 10% (v/v) FBS and 1% (v/v) pen–strep. All cell lines tested negative for mycoplasma contamination.

### Establishment of Stable Cell Lines

Overexpression and shRNA editing: BEST1 overexpressing lentivirus and ITPR1 knockdown lentivirus were generated by Tsingke Biotechnology Co., Ltd (Beijing, China). THP‐1 cells were infected with the lentivirus for 48 h, and the transfected cells were selected in 1 mg mL^−1^ puromycin.

CRISPR‐Cas9 gene editing: BEST1 CRISPR‐Cas9 lentivirus was generated by Tsingke Biotechnology Co., Ltd. The following sgRNA sequences to target human BEST1 were used: sgRNA‐1: 5′‐ CCAGCGTCACGTAGAAGCCT ‐3′; sgRNA‐2: 5′‐ CTGGTGTCGGGCTTCGTCGA ‐3′.

### Single‐Cell Gene Expression Analysis

This work obtained scRNA‐seq data from GSE139324 and GSE103322, which included monocytes, macrophages, and other immune cells isolated from HNSCC patients. Uniform Manifold Approximation and Projection for Dimension Reduction (UMAP) were applied to visualize inferred cell clusters. All the statistical analyses were conducted using R software (v4.0.4). In detail, the CD14^+^ monocytes cluster was identified with CD14 and LY*Z*, and the CD16^+^ monocytes cluster was identified with CD16 (FCGR3A) and MS4A7, and performed differentially expressed genes (DEGs) analysis in HNSCC patients and healthy donors.^[^ ^56^ ^]^ These DEGs expression were further analyzed on each cell cluster of each sample and captured BEST1 as a marker gene in peripheral blood monocytes of this HNSCC cohort dataset.

### Isolation of PBMC

Peripheral blood was obtained by venipuncture and collected into tubes containing EDTA coagulant. Blood was processed into PBMC by Ficoll density gradient centrifugation. Briefly, whole blood was diluted and layered over Ficoll (Solarbio), followed by centrifugation at 400 g for 30 min with the brake set to off. PBMC were then collected and washed in PBS.

### Flow Cytometry Staining and Analysis

After one wash with PBS, cells were incubated with appropriate dilutions of various combinations of the following antibodies for 30 min. Primary antibodies to cell surface markers directed against CD14 (17‐0149‐42), CD16 (12‐0167‐42), and BEST1 (PA5‐77290) were purchased from Invitrogen, VEGFR1 (13 687), and VEGFR2 (26 415) were purchased from Proteintech. The stained cells were acquired by a BD LSR II Flow Cytometer (BD Biosciences), and data generated were processed using FlowJo software.

### RNA Extraction and Real‐Time PCR

Total RNA was isolated using TRIzol reagent (Invitrogen). cDNA was synthesized from 2 µg RNA using a Quantscript RT kit (Takara). Relative gene expression was determined by real‐time PCR using the following primers:

BEST1
Forward – CTGGGCTTCTACGTGACGC
Reverse – TTGCTCGTCCTTGCCTTCG
CD80
Forward ‐AAACTCGCATCTACTGGCAAA
Reverse ‐GGTTCTTGTACTCGGGCCATA
CD86
Forward ‐CTGCTCATCTATACACGGTTACC
Reverse ‐GGAAACGTCGTACAGTTCTGTG
CD163
Forward ‐TTTGTCAACTTGAGTCCCTTCAC
Reverse ‐TCCCGCTACACTTGTTTTCAC
CD206
Forward ‐GGGTTGCTATCACTCTCTATGC
Reverse ‐TTTCTTGTCTGTTGCCGTAGTT
CCR2
Forward ‐TACGGTGCTCCCTGTCATAAA
Reverse ‐TAAGATGAGGACGACCAGCAT
ELK1
Forward – CAGCCAGAGGTGTCTGTTACC
Reverse – GAGCGCATGTACTCGTTCC
HIF1A
Forward – GAACGTCGAAAAGAAAAGTCTCG
Reverse – CCTTATCAAGATGCGAACTCACA
GAPDH
Forward ‐GGAGCGAGATCCCTCCAAAAT
Reverse ‐GGCTGTTGTCATACTTCTCATGG

### RNA Sequencing and Data Analysis

HOK and CAL27 cells were cultured with DMEM medium and then total RNA was purified using Trizol. RNA samples were sent to Shanghai Majorbio Bio‐pharm Technology Co., Ltd for library construction and sequencing. The data were analyzed on the online platform of Majorbio Cloud Platform (www.majorbio.com). Differential expressed genes between the HOK and CAL27 samples were obtained by using R package DESeq2 with filtering parameters of fold change above 2, adjusted *p* < 0.01, and average log2(TPM) in the high expression group above 0. Volcano plots were drawn using log2 (fold change) and ‐log10 (adjusted p), ceiled at 20 and 300, respectively.

### Differentiation of THP‐1 Cells and Coculture System

To generate THP‐1‐derived M1 and M2 macrophages, THP‐1 cells were treated with 100 nm phorbol 12‐myristate 13‐acetate (PMA) for 24 h, and then cultured with 100 ng mL^−1^ LPS and 20 ng mL^−1^ IFN‐*γ* (M1), or 20 ng mL^−1^ IL‐4 and 20 ng mL^−1^ IL‐13 (M2) for another 48 h.

For THP‐1 and tumor cells coculture, THP‐1 cells (in 0.4 mm transwell insert for 6‐well plate, 2 × 10^5^ cells per well) were treated with or without 100 nm PMA for 24 h, and cultured in fresh DMEM or MEM medium. The insert was then transferred onto the top of the CAL27 or FaDu cells (in 6‐well plate, 2 × 10^5^ cells per well) for 3 days.

For the production of the conditioned media from THP‐1‐derived macrophages, PMA was removed by thorough wash, and macrophages were further cultured in 2 mL fresh DMEM or MEM medium for another 24 h. Conditioned medium was collected and stored at −80 °C.

PMA and LPS were purchased from Sigma‐Aldrich. IFN‐*γ*, IL‐4, and IL‐13 were purchased from Peprotech.

### Plasma Membrane Protein Isolation

Plasma membrane proteins were extracted by Fractionation Kit according to the manufacturer's instructions (SM‐005, Invent). In brief, 1 × 10^7^ THP‐1 cells were first sensitized by buffer A before passing through the proprietary filter in a zigzag manner when high‐speed centrifugal force was applied, resulting in a cell lysate containing ruptured cell membranes. Then, after removing the intact nucleus by low‐speed centrifugation, the pellet obtained by prolonged high‐speed centrifugation was the total membrane protein fraction including plasma membrane (PM). The pellet was fully resuspended by buffer B and centrifuged to remove the organelle membranes pellet. As a result, PM was further separated by adding 8 volumes of PBS to adjust the supernatant density following by prolonged high‐speed centrifugation. PM fractions could be obtained at the completion of the protocol.

### Western Blotting

In brief, cells were collected using a scraper and washed once with cold PBS. The cells were then lysed in lysis buffer (50 mm Tris‐HCl, 250 mm NaCl, 5 mm EDTA, 50 mm NaF, 0.1% NP‐40) supplemented with 1% protease inhibitor cocktail. Equal amounts of proteins were size‐fractionated by 7.5%–15% SDS‐PAGE. Primary antibodies against p‐MEK1/2 (9154), p‐ERK1/2 (4370), ERK1/2 (4695), p‐AKT (Ser473) (4060), and AKT (4691) were purchased from Cell Signaling Technology, MEK1/2 (11 049) was purchased from Proteintech, Na^+^‐K^+^ ATPase (ab76020) was purchased from Abcam, and *β*‐actin (A5441) was purchased from Sigma‐Aldrich. At least three independent experiments were performed.

### Dual‐Luciferase Reporter Assay

THP‐1 cells were grown according to standard protocols. pGL4.10 vectors fused with a fragment spanning from −2000 to 0 of the TSS of the BEST1 genomic sequence with or without truncated deletions or mutation were electronically transfected into THP‐1 cells (according to BTX ECM 830 electroporation protocol). The Luciferase Reporter Assay System (E1500, Promega) was used according to the manufacturer's instructions.

### Cell Immunofluorescence

THP‐1 cells were fixed with 4% paraformaldehyde, and then blocked with 3% BSA/PBS. The cells were incubated with anti‐BEST1 and anti‐Na^+^‐K^+^ ATPase (ab283318) overnight at 4 °C. Alexa Fluor488‐conjugated Goat anti‐rabbit antibody (ZF‐0511, ZSGB‐BIO) and Alexa Fluor594‐conjugated Goat anti‐mouse antibody (ZF‐0513, ZSGB‐BIO) were used as the secondary antibody. The samples were observed under a Leica TSC‐SP2 Microsystems.

### Staining of Tissue Sections

Sectioning and immunohistochemical (IHC) staining of formalin fixed, paraffin‐embedded (FFPE) HNSCC specimens was performed by the standard protocols. All sections were 5 mm thick. Briefly, sections were deparaffinized through xylenes and graded ethanol, and antigen retrieval was performed in Tris/EDTA buffer at pH 9.0. For multiple immunofluorescence staining, PANO 4‐plex IHC kit (PANOVUE) was used according to the manufacturer's instructions. The samples were then observed under a Leica TSC‐SP2 Microsystems. Primary antibodies against CD14 (ab133335), CD68 (ab213363) were purchased from Abcam.

### Determination of Intracellular Ion Concentration

The calcium ion fluorescent probe Fluo‐3 AM (S1056, Beyotime Institute of Biotechnology, Shanghai, China) was excited at a wavelength of 488 nm and emitted light was collected at a wavelength of 525 nm. The chloride ion fluorescent probe *N*‐(ethoxycarbonylmethyl)‐6‐methoxyquinolinium bromide (MQAE) (S1082, Beyotime) was excited at a wavelength of 355 nm, and emitted light was collected at a wavelength of 460 nm.

THP‐1 cells medium was replaced with a culture medium containing 5 µm Fluo‐3 AM or 5 mm MQAE, and the cells were cultured in a 37 °C incubator for 1 h. The cells were washed and then incubated for another 30 min. The cells were treated with VEGF‐A at the concentration of 100 ng mL^−1^ and the fluorescence signals were observed every 20 s for 10 or 15 min with NikonA1 Live‐Cell Super‐Resolution Imaging System.

### Enzyme‐Linked Immunosorbent Assay

The levels of VEGF‐A, IL‐1*α*, IL‐1*β*, IL‐6, and IL‐8 in cell culture supernatant were detected by Enzyme‐Linked Immunosorbent Assay (ELISA) kits (CHE0043, CHE0101, CHE0001, CHE0009, CHE0011, and 4A Biotech) according to the manufacturer's instructions.

After THP‐1 cells were treated with or without 100 ng mL^−1^ VEGF‐A for 15 min, hypotonic lysate was applied to lysate the cells, and supernatant was obtained after centrifugation (1000×g, 10 min). The level of IP_3_ in cell lysates was detected by ELISA kit (ml060362, MLBio) according to the manufacturer's instructions.

### Time‐Lapse Imaging

48 h before the experiment, THP‐1 cells were plated at the low confluence (5000 cells per well) in a 24‐well plate. On the day of experiments, media was changed 1 h the beginning of experiments. Time‐lapse series were recorded using a Cellomics HCS Studio 3.0. Acquisition was performed over a 12 h period with an image every 30 min. For tracking cells, 2 individual cells in each field were manually tracked using the MTrack2 plugin of ImageJ. For measuring cell migration, the total distance traveled from each cell was measured.

### Transwell Migration Assay

Cell migration assays were performed in 24‐well transwell plates with 8 µm polyethylene terephthalate membrane filters (Corning) separating the lower and upper culture chambers. THP‐1 were seeded in the upper chamber at 1 × 10^6^ cells per well in DMEM with 1% FBS. The bottom chamber contained DMEM with 10% FBS. THP‐1 cells were allowed to migrate for 24 h. After the incubation period, the filter was removed, and non‐migrant cells on the upper side of the filter were detached using a cotton swab. Filters were fixed with 4% formaldehyde for 15 min, and the cells located in the lower filter were stained with 0.1% crystal violet for 20 min and counted in three random fields.

### CCK‐8 Assay

Equal numbers of FaDu cells (5000 per well) were seeded into a 96‐well plate 24 h before experimentation. Cells were treated with different conditioned mediums for 48 h. After treatment, CCK‐8 (CK001, LabLead) was added to the 96‐well plate and incubated at 37 °C for 1 h. The absorbance of each sample was read at 450 nm.

### Colony Formation

FaDu cells were seeded in the 6‐well plates at 500 cells per well and cultured with different conditioned mediums for 14 days, and then treated with the 0.5% crystal violet solution for staining in 4% paraformaldehyde. Finally, colonies were counted manually.

### EdU Incorporation Assay

For EdU analysis, FaDu cells were cultured with 10 µm EdU for 2 h and then treated with 4% paraformaldehyde for fixation. After permeabilization, EdU Imaging Kits (Cy3) (K1075, Apexbio) was used according to the manufacturer's instructions. Hoechst 33 342 solution was used to counterstain cell nuclei. Proliferative cells were determined under the confocal microscope.

### Animal Studies

For tracking the monocytes in circulation, FaDu cells (2 × 10^6^) were delivered into 5‐week‐old male BALB/c nude mice via hypodermic injection. After 2 weeks, THP‐1 cells (1 × 10^6^), labeled with DiR dye, were injected via tail vein. The bioluminescent images were examined by using an IVIS LuminaIIin vivo bioluminescence imaging system (Caliper Life Science) on Day 1 after injection.

For tumorigenesis, 4 × 10^5^ THP‐1 cells were combined with 2 × 10^6^ FaDu cells at a 1:5 E:T ratio and co‐implanted subcutaneously to the flanks of the 5‐week‐old male BALB/c nude mice. Tumor volume was monitored by caliper measurements. Mice were euthanized to harvest tumors after 4 weeks after tumor implantation.

### Statistical Analysis

The data represent the mean ± S.D. of at least two independent experiments. Statistical analyses were performed by GraphPad Prism 8. The differences between two groups were assessed by unpaired two‐tailed Student's *t‐*tests. Multi‐group comparisons were analyzed by one‐way or two‐way ANOVA with Tukey's post hoc test. *p* < 0.05 was considered statistically significant (*p < 0.05, **p < 0.01, and ***p < 0.001).

## Conflict of Interest

The authors declare no conflict of interest.

## Supporting information

Supporting Information

## Data Availability

The data that support the findings of this study are available from the corresponding author upon reasonable request.
