# Supplementary material for: BEST1 Positive Monocytes in Circulation: Visualize Intratumoral Crosstalk between Cancer Cells and Monocytes
Source: Adv Sci (Weinh). 2023 Apr 23;10(17):2205915. doi: 10.1002/advs.202205915 (PMC10265052; doi:10.1002/advs.202205915)
Supplement: Supplementary file 1 — Supporting Information [file ADVS-10-2205915-s001.pdf]

ADVANCED SCIENCE
Open Access

Supporting Information

for *Adv. Sci.*, DOI 10.1002/advs.202205915

BEST1 Positive Monocytes in Circulation: Visualize Intratumoral Crosstalk between Cancer Cells and Monocytes

Luyao Zhang, Yiran Wang, Wei Yuan, Changming An, Qin Tan and Jie Ma**

Title:

BEST1 Positive Monocytes in Circulation: Visualize Intratumoral Crosstalk between Cancer Cells and Monocytes

Authors:

*Luyao Zhang[1], Yiran Wang[1], Wei Yuan[2], Changming An[3], Qin Tan[1, *], Jie Ma[1, *]*

Affiliations:

L. Zhang, Y. Wang, Q. Tan, J. Ma

Center of Biotherapy

Beijing Hospital

National Center of Gerontology

Institute of Geriatric Medicine

Chinese Academy of Medical Sciences

Beijing, 100730, P. R. China

Email: qin_tan@bjmu.edu.cn；majie4685@bjhmoh.cn

W. Yuan

State Key Laboratory of Molecular Oncology

National Cancer Center/ National Clinical Research Center for Cancer/Cancer Hospital

Chinese Academy of Medical Sciences & Peking Union Medical College

Beijing, 100021, P. R. China

C. An

Department of Head and Neck Surgery

National Cancer Center/ National Clinical Research Center for Cancer/Cancer Hospital

Chinese Academy of Medical Sciences & Peking Union Medical College

Beijing, 100021, P. R. China

Supporting Information

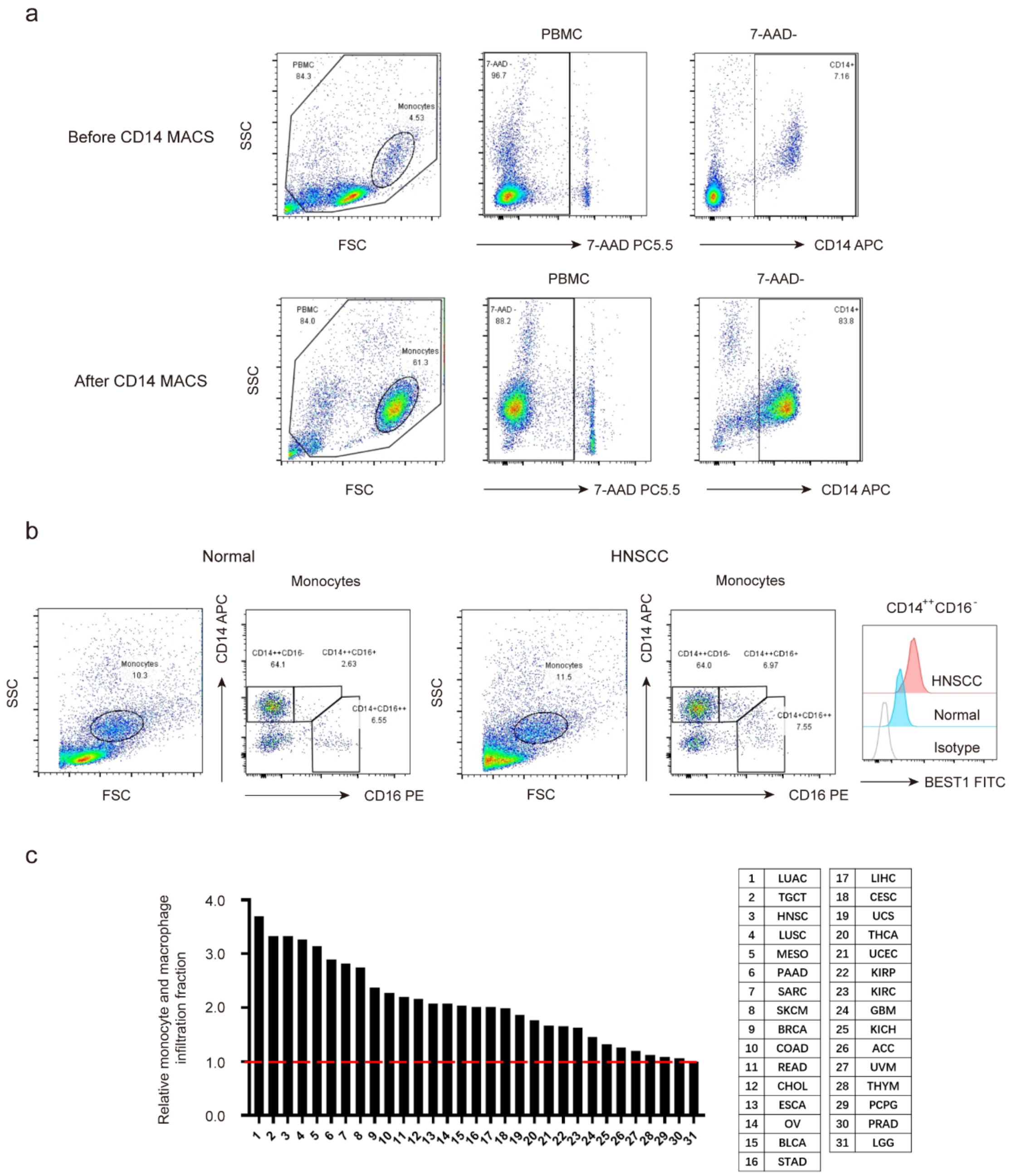

Figure S1. BEST1 is up-regulated in peripheral monocytes of patients with high monocyte/macrophage infiltration tumor types.

a) Representative plots showing the $CD14^+$ monocytes among PBMC before and after $CD14^+$ MACS. b) Representative plots and histograms showing the monocytes subpopulations and BEST1 expression of $CD14^{++}CD16^-$ monocytes in peripheral blood

from patients with HNSCC and healthy donors. c) Monocyte and macrophage infiltration in patient samples from The Cancer Genome Atlas (TCGA) assessed by CIBERSORT.

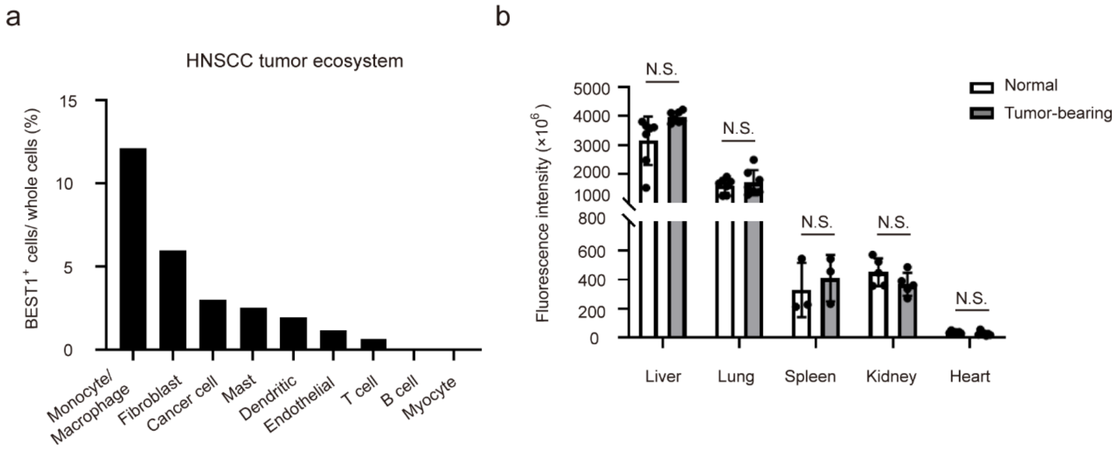

Figure S2. BEST1 is expressed on tumor-infiltrated monocytes/macrophages.

a) BEST1 positive ratio of immune cell subsets in HNSCC ecosystem using scRNAseq analysis. b) Fluorescence value of each organ of normal and tumor-bearing mice 24 h after DiR labeled THP-1 injection (n = 5 mice per group from one experiment representative of two independent experiments). Data are represented as mean ± SD. Statistical significance is indicated by N.S. = not significant; Student's t-test (b).

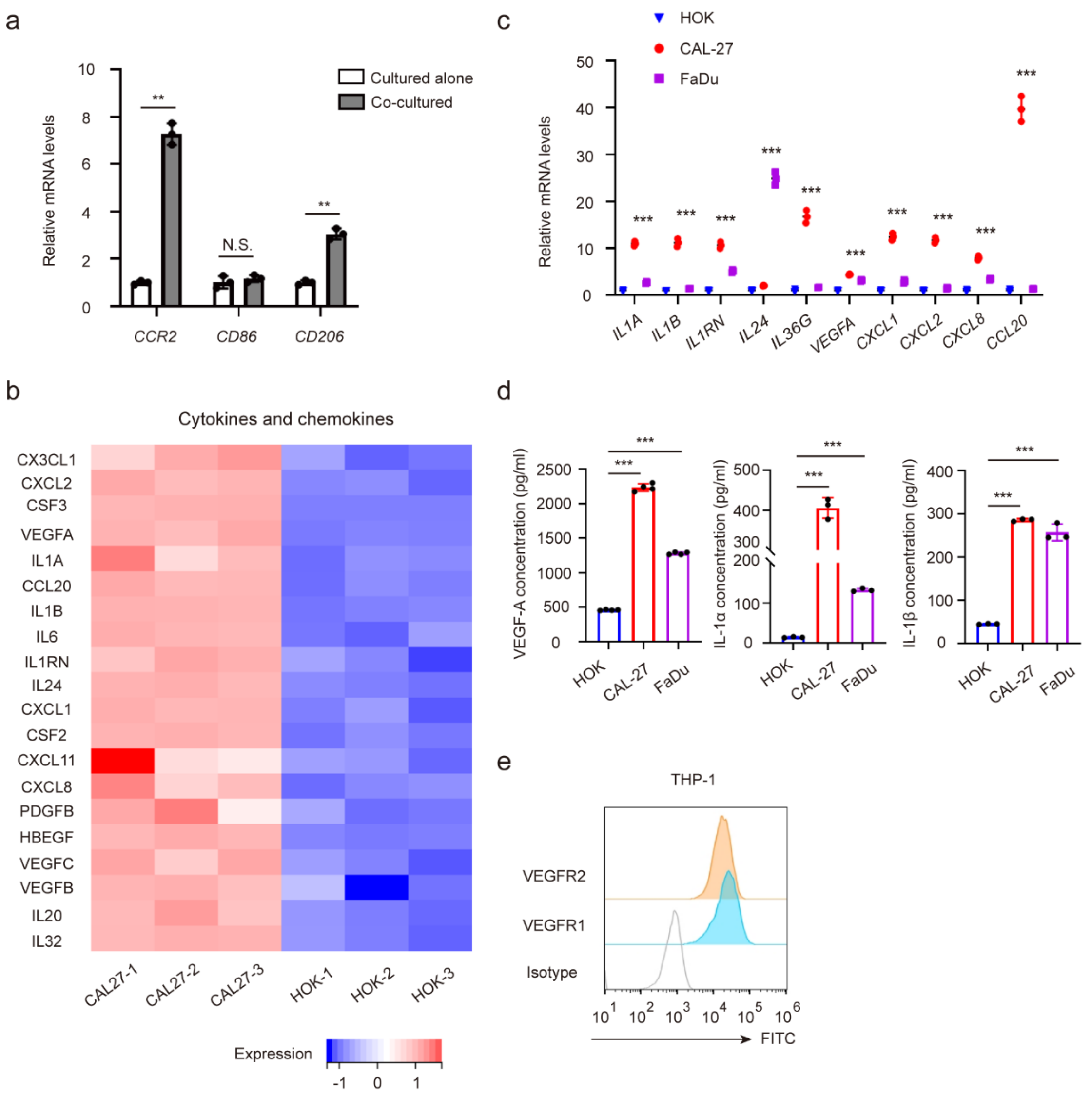

Figure S3. VEGF-A in tumor microenvironment increases BEST1 expression.

a) Relative *CCR2*, *CD86*, and *CD206* mRNA levels in Mφ cocultured with CAL27 for 3 days. The data are normalized to GAPDH (n=3 per group from one experiment representative of three independent experiments). b) Heatmap of “cytokines and chemokines” genes regulated in the same direction of CAL27 cells. c) Relative mRNA levels of cytokines in HOK, CAL27, and FaDu (n=3 per group from one experiment representative of three independent experiments). d) VEGF-A, IL-1α, and IL-1β levels in supernatant from HOK, CAL27, and FaDu detected by ELISA (n=4 per group from one experiment representative of two independent experiments). e) Flow cytometry of

VEGFR1 and VEGFR2 expression on THP-1 is shown. Data are represented as mean ± SD. Statistical significance is indicated by $^{**}P < 0.01$, $^{***}P < 0.001$; Student's t-test (a); one-way ANOVA (c), (d).

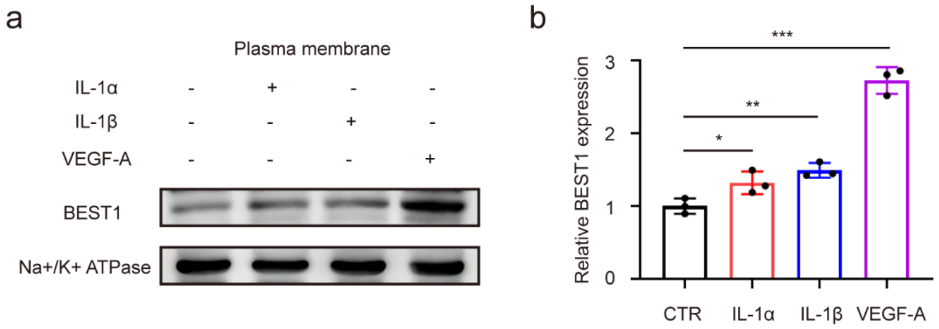

Figure S4. BEST1 localizes on the plasma membrane of THP-1.

a, b) Representative western blot (a) and associated quantifications (b) of BEST1 expression on the plasma membraned of THP-1 treated with 10 ng/ml cytokines for 24 h, respectively (n=3 per group from one experiment representative of two independent experiments). Data are represented as mean ± SD. Statistical significance is indicated by **P* < 0.05, ***P* < 0.01, ****P* < 0.001; one-way ANOVA (b).

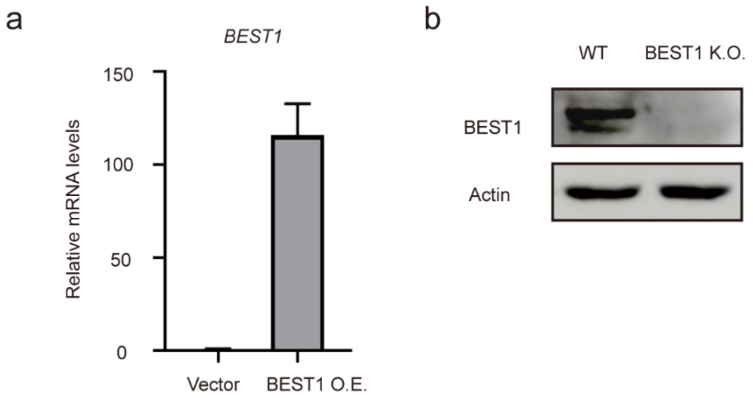

Figure S5. VEGF-A in tumor microenvironment increases BEST1 expression.

a) Relative BEST1 mRNA levels in THP-1 with Vector and BEST1 O.E. The data are normalized to GAPDH (n=3 per group from one experiment representative of three independent experiments). b) Representative Western blots show BEST1 levels in THP-1 with WT and BEST1 K.O. (n=3 per group from one experiment representative of three independent experiments).

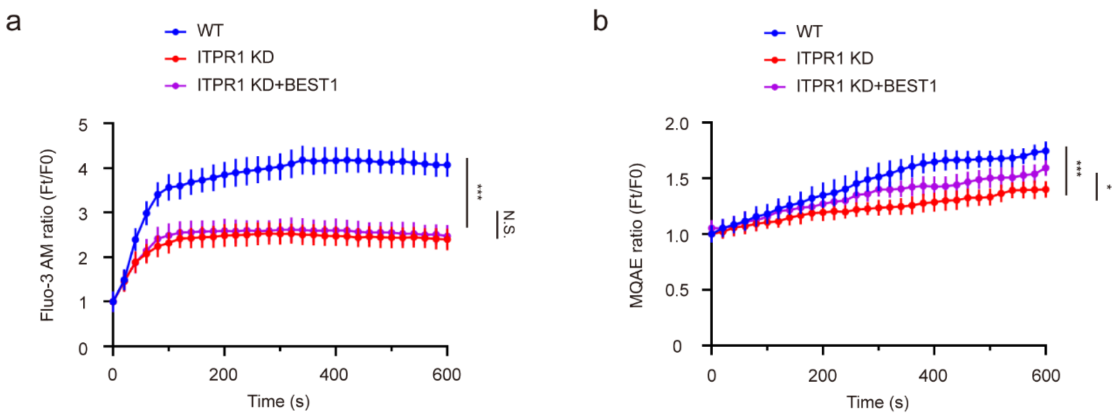

Figure S6. Activation of BEST1 by IP_3-mediated Ca^{2+} release from the ER.

a, b) Quantification of Fluo-3AM (a) and MQAE (b) in VEGF-A-treated WT, ITPR1 KD, and ITPR1 KD with BEST1 overexpression THP-1, respectively (n= 3 per group from one experiment representative of two independent experiments). Data are represented as mean ± SD. Statistical significance is indicated by ***$P < 0.001$; two-way ANOVA with Tukey's test (a, b).

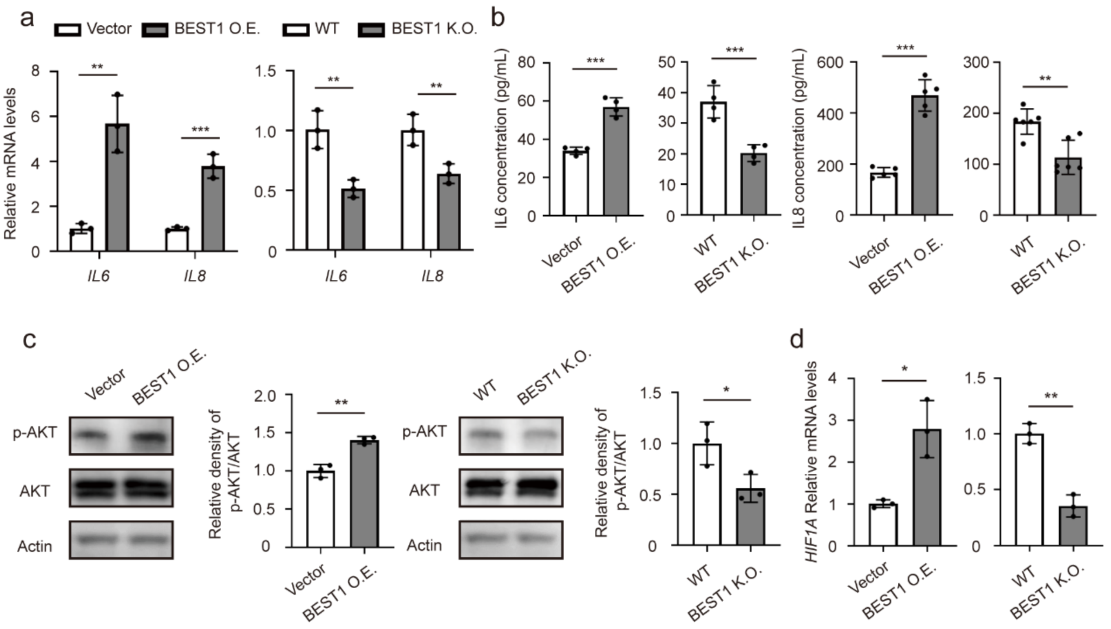

Figure S7. BEST1 activates AKT-HIF-1α to upregulate IL-6 and IL-8.

a) Relative *IL6* and *IL8* mRNA levels in Mφ with Vector and BEST1 O.E. (left), or WT and BEST1 K.O. (right). The data are normalized to GAPDH (n=3 per group from one experiment representative of three independent experiments). b) IL-6 and IL-8 levels in supernatant from Mφ with Vector and BEST1 O.E. (left), or WT and BEST1 K.O. (right) detected by ELISA (n=5-6 per group from one experiment representative of two independent experiments). c) Representative Western blot and the associated quantifications show phosphorylated AKT levels in THP-1 with Vector and BEST1 O.E., or WT and BEST1 K.O. (Data are representative of three independent experiments). d) Relative *HIF1A* mRNA levels in THP-1 with Vector and BEST1 O.E., or WT and BEST1 K.O. The data are normalized to GAPDH (n=3 per group from one experiment representative of three independent experiments). Data are represented as mean ± SD. Statistical significance is indicated by $*P < 0.05$, $**P < 0.01$, $***P < 0.001$; Student's t-test (a), (b), (c), (d).

Table S1. Differentially Expressed Genes in monocytes (HNSCC patients/health donors)

Gene name	logFC	logCPM	PValue	FDR
BEST1	1.524922887	9.246232132	8.73E-35	3.39E-31
IGHA1	1.818083348	8.986664991	3.46E-28	1.01E-24
IGLC2	1.49642736	8.777067497	1.16E-25	2.71E-22
RP11-1143G9.4	1.768930369	9.403074615	1.13E-22	2.19E-19
IFI44L	1.257883948	8.860121536	3.67E-16	2.85E-13
HLA-DQA2	1.323902961	8.886765219	1.84E-15	1.13E-12
LGALS2	1.428710498	8.909850101	1.07E-14	4.80E-12
FOSB	1.129759752	8.793417708	6.70E-12	2.11E-09
MX1	1.028875497	8.822910919	2.68E-11	7.62E-09
